# 

**Published:** 1861-03

**Authors:** 


AMERICAN.

Researches on the Venom of the Rattlesnake; with an Investigation of the Anatomy and Physiology of the Organs Concerned. By S. W. Mitchell, M.D., etc. 4to., pp. 145. Smithsonian Institution: Washington, 1861. (From the author.)

Proceedings of the Pathological Society of Philadelphia. Vol. i. 8vo., pp. 307. Philadelphia: J. B. Lippincott & Co., 1860. (From the secretary.)

Transactions of the American Medical Association. Vol. xiii. 8vo., pp. 930. Philadelphia, 1860. (From the secretary.)

A Practical Treatise on Phthisis Pulmonalis; embracing its Pathology, Causes, Symptoms, and Treatment. By L. McLawson, M.D., etc. 8vo., pp. 557. Cincinnati: Rickey, Mallory & Co., 1861. (From the publishers.)

A Catalogue of the Pathological Cabinet of the New York Hospital. By Robert Ray, Jr., M.D. 8vo., pp. 364. New York: S. S. & W. Wood, 1860. (From the hospital.)

The Institutes of Medicine. By Martyn Paine, M.D., etc. 6th edition. 8vo., pp. 1109. New York: Harper & Brothers, 1860. (From the author.)

Theory and Practice of the Movement Cure. By Charles Fayette Taylor, M.D. 12mo., pp. 295. Philadelphia: Lindsay & Blakiston, 1861. (From the publishers.)

Electro-Physiology and Electro-Therapeutics; showing the Best Methods for the Medical Uses of Electricity. By A. C. Garratt, M.D., etc. 2d edition. 8vo., pp. 716. Boston: Ticknor & Fields, 1861. (From the author.)

Diphtheria; its Nature and Treatment, with an Account of the History of its Prevalence in Various Countries. By D. D. Slade, M.D. 8vo., pp. 85. Philadelphia: Blanchard & Lea, 1861. (From the publishers.)

Report on the Importance and Economy of Sanitary Measures to Cities. By John Bell, M.D. 8vo., pp. 243. New York: Jones & Co., 1860. (From the author.)

Proceedings and Debates of the Fourth National Quarantine and Sanitary Convention, held in the City of Boston, June, 1860. 8vo., pp. 282. Boston, 1860. (From the secretary.)

To What Affections of the Lungs does Bronchitis give Origin? Prize essay. By D. D. Slade, M.D. 8vo., pp. 75. (From the author.)

On Uræmic Intoxication. By W. A. Hammond, M.D., etc. Reprint, pp. 31. Philadelphia, 1861. (From the author.)

Contributions to the Anatomy of the Spinal Cord. By J. B. Trask, M.D. 8vo., pp. 16. San Francisco, 1861. (From the author.)

Report of Stone's Infirmary, for the Year Ending August 31st, 1860. By T. G. Richardson, M.D. Reprint, pp. 24. New Orleans, 1861. (From the author.)

Report of the Kentucky Eastern Lunatic Asylum, for the Year 1860. Pamphlet, pp. 15. Frankfort, 1861. (From Dr. W. S. Chipley.)

Twenty-second Annual Report of the Central Ohio Lunatic Asylum, for the Year 1860. Pamphlet, pp. 28. Columbus, 1861. (From Dr. Hills.)

First Annual Report of the Superintendent of Clifton Hall, a Private Hospital for the Insane, for 1860. Pamphlet, pp. 8. Philadelphia, 1861. (From Dr. Given.)

A Case of Fracture of the Os Innominatum, and Death in Connection with the Administration of Sulphuric Ether. By W. H. Mussey, M.D. Reprint, pp. 8. Cincinnati, 1861. (From the author.)

FOREIGN.

On Diphtheria. By E. H. Greenhow, M.D. etc. 8vo., pp. 160. Baillière Brothers: New York, 1861. (From the publishers.)

A Hand-Book of Hospital Practice; or an Introduction to the Practical Study of Medicine at the Bedside. By R. H. Lyons, M.D., etc. 12mo., pp. 185. New York: S. S. & W. Wood, 1861. (From the publishers.)

Anatomy of the Arteries of the Human Body, Descriptive and Surgical, with the Descriptive Anatomy of the Heart. By J. H. Power, M.D., etc. 12mo., pp. 374. Dublin: Fannin & Co., 1860.

Conférences de Clinique Chirurgicale faites à L'Hotel-Dieu, pendant l'Année 1858–1859. Par M. A. C. Robert. 8vo., pp. 542. Paris: Germer Baillière, 1860.

The Science and Art of Surgery. By John E. Erichsen. 3d edition. 8vo., pp. 1167. London: Walton & Maberly, 1861.

Des Tumeurs du Corps Thyroide. Par C. Houel, M.D. 4to., pp. 99. Paris: Martinet, 1860.

The Principles and Practice of Surgery. By W. Pirrie, F.R.S.E. 8vo., pp. 878. 2d edition. London: John Churchill, 1860.

